# Nine weeks of high-intensity indoor cycling training induced changes in the microbiota composition in non-athlete healthy male college students

**DOI:** 10.1186/s12970-021-00471-z

**Published:** 2021-12-18

**Authors:** Sabrina Donati Zeppa, Stefano Amatori, Davide Sisti, Marco Gervasi, Deborah Agostini, Giovanni Piccoli, Valerio Pazienza, Pietro Gobbi, Marco B. L. Rocchi, Piero Sestili, Vilberto Stocchi

**Affiliations:** 1 Department of Biomolecular Sciences, University of Urbino Carlo Bo Piazza Rinascimento 7, 61029 Urbino, Italy; 2 Division of Gastroenterology “Casa Sollievo della Sofferenza” Hospital, 71013 San Giovanni Rotondo, Italy; 3 Università Telematica San Raffaele, 00166 Rome, Italy

**Keywords:** gut microbiota, physical exercise, diet habits, high-intensity interval exercise

## Abstract

**Background:**

The gut microbiota constitutes a dynamic microbial system constantly challenged by environmental conditions, including physical exercise. Limited human studies suggest that exercise could play a beneficial role for gut health, increasing microbial diversity, even if the effects of exercise on gut microbial microorganisms depends on its intensity and duration. This study aimed to investigate the effects of nine weeks of high-intensity interval exercise on gut microbiota composition in healthy young adults.

**Methods:**

The gut microbiota composition of seventeen healthy male college students was analysed before and after nine weeks of high-intensity interval cycling training by 16S rRNA amplicon sequencing. PERMANOVA for repeated measures was used to test pre-post differences in the relative abundance of all taxonomic levels, and correlations between variations in microbial composition and physical and dietary features were also assessed.

**Results:**

Physical exercise induced changes in microbiota composition, at all taxonomic levels analysed (phyla: F _[1, 32__]_=3.97, *p*=0.029; classes: F _[__1, 32__]_=3.39, *p*=0.033, orders: F _[__1, 32__]_=3.17, *p*=0.044, families: F _[__1, 32__]_=1.54, *p*=0.037, genera: F _[__1, 32__]_=1.46, *p*=0.015, species: F _[__1, 32__]_=1.38, *p*=0.007). Conversely, no differences were found between pre and post-training conditions for microbial community richness (Chao1: V=105, *p*=0.06) or diversity (Shannon index: V=62, *p*=0.52; Simpson index: V=59, *p*=0.43). Changes in the relative abundance of eighteen genera were correlated to changes of twenty environmental factors grouped in physical features, sport-related features, and dietary features.

**Conclusions:**

Nine weeks of high-intensity exercise induced modifications in gut microbiota composition in healthy male college students, shifting the gut microbial population towards a healthier microbiome with benefit to human health in general.

**Supplementary Information:**

The online version contains supplementary material available at 10.1186/s12970-021-00471-z.

## Background

The human gut microbiota is composed of over a hundred trillion microbial cells, and its association with human health has been increasingly studied in the last decades [1, 2]. The gut microbial community significantly influences the host's metabolism and immune system, representing a risk or protective factor to several immune-allergic and metabolic disturbances [3, 4]. Its composition is highly subjective, and it is associated with environmental and behavioural factors, such as age, diet [1], supplementation [5] and physical exercise [6]. The latter was linked with positive changes in the gut microbiota diversity and community, both in animal and human studies [7, 8]. Among humans, positive correlations were reported between bacterial diversity, butyrate-producing bacteria and cardiorespiratory fitness (VO_2max_) [9], and higher turnover of carbohydrates and proteins, and concentrations of short-chain fatty acids in athletes compared to sedentary controls [10]. An observational study conducted on elite male rugby players showed that athletes have a higher abundance of *Firmicutes* and lower levels of *Bacteroidetes* than non-athletes healthy controls [11]. In a recent study, higher microbial diversity, different taxonomic and functional composition, and a strong association with athletes performance in elite athletes with respect to non-elite was reported when faecal samples of nineteen individuals pertaining to three cohorts (adult elite, youth elite and non-elite athletes) were compared [1]. A review published by Mitchell and colleagues in 2019 [12] reported only one longitudinal intervention study [13] where dietary intake and exercise were monitored, and participants tested pre and post-training. Indeed, an increase in beta-diversity after six weeks of endurance training in obese individuals was observed [13]. Authors controlled the dietary intake for three days before the stool collection to minimise the likely confound of differences in dietary intake on microbial composition; nevertheless, diet monitoring for a more extended period, before and during the training, was missing. This point should be crucial in these intervention studies, as changes in microbiota composition could not be attributed exclusively to the exercise but also to changes in the dietary intake, which was shown to be influenced by exercise itself [14]. Other studies were recently published investigating the effect of light-to-moderate intensity exercise training on changes in microbiota profile in overweight adults [15], women [16], and obese children [17]. Rettedal et al. [18] investigated the effect of a short-term high-intensity interval training period on the gut bacterial composition of lean and overweight men, failing to find changes neither in diversity nor composition of the microbiota. Considering the above-presented studies, a systematic review recently published [19] have pointed out the need for longer duration and higher intensity training studies to analyse significant taxonomic changes in microbiota composition further.

Hence, this study aimed to investigate the effects of 9-week high-intensity interval exercise on gut microbiota composition in active, healthy young adults. To the authors' knowledge, this is the first training study with a structured exercise period and daily monitoring of training and dietary intake.

## Methods

### Participants

Eighteen healthy college students (22±2 years, 175.7±6.7 cm, 69.1±10.1 kg, 22.3±2.7 kg/m^2^) were recruited. The inclusion criterion was to be sedentary: by completing a specific survey, subjects declared not performing more than one 60 min low-intensity training session per week in the three months before the start of the study. Baseline VO_2max_ values confirmed the low training level of the participants. Exclusion criteria were musculoskeletal injuries, major cardiovascular diseases, upper respiratory infections, smoking in the past three months. The participants were advised to maintain their dietary routine and to refrain from other sports activities except the sessions scheduled for the experimental design. After a medical health screening, all participants signed a written informed consent to take part in the study, which was approved by the Ethics Committee of the University of Urbino Carlo Bo, Italy (no. 02/2017, approved on July 10, 2017) and was conducted in accordance with the Declaration of Helsinki for research with human volunteers (1975).

### Study design

This was a longitudinal training study in which gut microbiota was analysed before and after nine weeks of high-intensity interval cycling training. Before and after the training period, body composition and maximal oxygen consumption (VO_2max_) were assessed. Participants arrived at the laboratory in the morning, after six or more hours of fasting. Body composition was measured through electrical bioimpedance (BIA101 Sport Edition, Arkray, Kyoto, Japan). They were asked to drink enough fluids over the 24h prior to the measurement to ensure a normal hydration state, and to abstain from alcohol and caffeine in the 8 hours before the test. After completing the body composition assessment, subjects received a standardised breakfast consisting of jam tart (~135 g per serving) and 400 ml of fruit juice. Two hours later, they started the testing session. Each participant performed a maximal graded exercise test to assess VO_2max_, peak power output (W_peak_) and power at lactate thresholds (W_LT1_ and W_LT2_). The test was performed on an electronically-braked ergometer (SRM Italia, Lucca, Italy) at 75W, and power was increased by 25W every 3 min until volitional exhaustion. Oxygen consumption was monitored breath-by-breath using a wearable metabolic cart (Cosmed K4b2, COSMED, Rome, Italy), and blood lactate was measured in the last 15 seconds of each stage from the tip of the index finger using a blood lactate meter (Lactate-Pro, Arkray, Kyoto, Japan). For more detailed information, see Gervasi et al. [20].

#### Training characteristics

Thirty-six indoor cycling training sessions during nine weeks were performed. The training program consisted of three mesocycles (three weeks each), on which both frequency and duration of the sessions increased: 3 × 55 min sessions per week in the first mesocycle, 4 × 60 min sessions per week in the second mesocycle, 5 × 70 min sessions per week in the last mesocycle. Each session was structured with a warm-up, a high-intensity interval exercise and a cool down. Intervals were performed at different intensities and durations to achieve 20% of the total training volume at an intensity above the second lactate threshold (LT_2_). Heart rate (HR) was recorded during each session, and HR values were used by the participants to match the requested intensity on each interval, according to their individual training zones defined with the incremental test.

#### Dietary monitoring

Diet was daily monitored from two weeks before the beginning of the training period until the end of the study, and it was performed by call interviews each day after dinner. Information about food and drink consumed were collected and then processed through MètaDieta software (METEDA Srl, San Benedetto del Tronto, Italy), and the following variables were considered for the analyses: total energy intake, macronutrients quantity, starch, soluble and insoluble fibres, saturated, monounsaturated, polyunsaturated fat, Omega-3, Omega-6, iron, vitamin A, C, D, E and amino acids (alanine, valine, isoleucine, leucine, tryptophan).

### Faeces sample collection and DNA extraction

Faeces samples were collected at baseline and after the nine weeks of training. Faecal samples were collected in a sterilized 50 mL tube without additives, ethanol or stabilizing solutions, immediately stored and placed on frozen packages and delivered to the laboratory to be stored at -80 °C within two hours. For 16SrDNA sequencing, the total microbial DNA of the samples was extracted using the QIAamp PowerFecal DNA Kit (Qiagen) following the manufacturer’s protocol. After assessing DNA concentration and purity, samples were stored at -80° until processing.

### 16S rRNA gene sequence data processing

The V3–V4 hypervariable regions of the bacterial 16S ribosomal RNA gene were amplified for each DNA sample using the universal primers, as reported in Klindworth et al. [21]. Agencourt AMPure XP beads (Beckman Coulter, Milan, Italy) were used to purify PCR amplicons. The latter was used for a second PCR to barcode the libraries using the Illumina dual-index system (Nextera XT Index Kit, Illumina Inc., San Diego, CA, USA) necessary for multiplexing. The purified DNA products were then subjected to a further PCR to attach dual Illumina indices (Nextera XT Index Kit, Illumina Inc., San Diego, CA, USA) necessary for multiplexing. According to the manufacturer's instructions, paired-end sequencing (2 × 300 cycles) was carried out on an Illumina MiSeq instrument (Illumina Inc.). Sequences were demultiplexed based on index sequences, and FASTQ files were generated. Sequences were then analysed using QIIME 1.9.1 software and following the pipeline [22]. Sequences were quality filtered to remove short and long sequences (truncated sequence should be > 75 bases long), uncorrected barcodes, ambiguous bases, and sequences with primer mismatches. Chimeric sequences were identified and removed from all downstream analyses. To that end, both de novo and reference chimaeras were identified and removed employing usearch 6.1 and the GreenGenes database (v. 105 13-8, 97% identity). The latter was also used for open reference OTU (Operational Taxonomic Units) picking and taxonomic assignment through UCLUST.

### Statistical analyses

One subject was excluded from the analyses because of faecal sample collection errors. Alpha diversity indexes (Chao1, Shannon H and Simpson) were calculated using the *diversity* function of the *vegan* R package [23], both in pre and post-training conditions. Differences among the pre and post-training diversity indexes were tested using a Wilcoxon signed-rank test for paired data. Bray-Curtis dissimilarity was calculated using *vegdist* R function; Permutational Analysis of Variance (PERMANOVA) for repeated measures was used to test pre-post differences in the relative abundance of all taxonomic levels (phyla, classes, orders, families, genera and species), using *adonis* R function with subjects’ ID as strata for pairing. Post-hoc comparisons at phylum and genus levels were performed using a Wilcoxon test, and effect sizes were calculated for all comparisons using the *wilcox_effsize* function of *rstatix* [24] and *coin* R packages [25]. Common interpretations of Wilcoxon effect sizes (r) are: 0.10 - 0.3 (small effect), 0.30 - 0.5 (moderate effect) and >= 0.5 (large effect) [26]. A ternary plot was built to visualise the co-variations of the relative abundances in the three most dominant phyla, using the *ggtern* R package [27]. Pre-post comparisons between all phyla present in our sample were graphically reported as box plots, using the *ggplot2* R package [28]. Genera were then filtered for a relative abundance higher or equal to 1%, in pre or post-training conditions, for the subsequent analyses. A correlation matrix was calculated on the post-pre differences of the relative abundance of the 29 remaining genera, and it was graphically represented on a non-parametric correlation plot, all using the *corrplot* R package [29]. To assess post-pre changes in global genera relative abundances, a non-metric dimensional scaling (nMDS) was performed, with the Bray-Curtis distance, using the *metaMDS* function of the *vegan* R package [23]. Finally, from an exploratory point of view, to assess correlations between variations in microbial composition and physical and dietary features, a circular correlation plot was built, using the *chordDiagram* function of the *circlize* R package [30]. Only significant correlations (Spearman r ≥ 0.485, for n=17; p≤0.05, two-tailed) are reported in the circle plot for visual clarity. Statistical analyses were performed using R Studio 1.4; the significance threshold was fixed at the standard level of 0.05.

## Results

### Exercise shapes gut microbiota composition without affecting richness and diversity indices

Seventeen healthy male subjects completed thirty-six indoor cycling training sessions during nine weeks. Training sessions were: 3 × 53.1±1.3min sessions per week for the first three weeks, 4 × 59.1±1.2min sessions per week for the second three weeks, 5 × 68.2±1.4min sessions per week for the last three weeks.

**Fig. 1:**
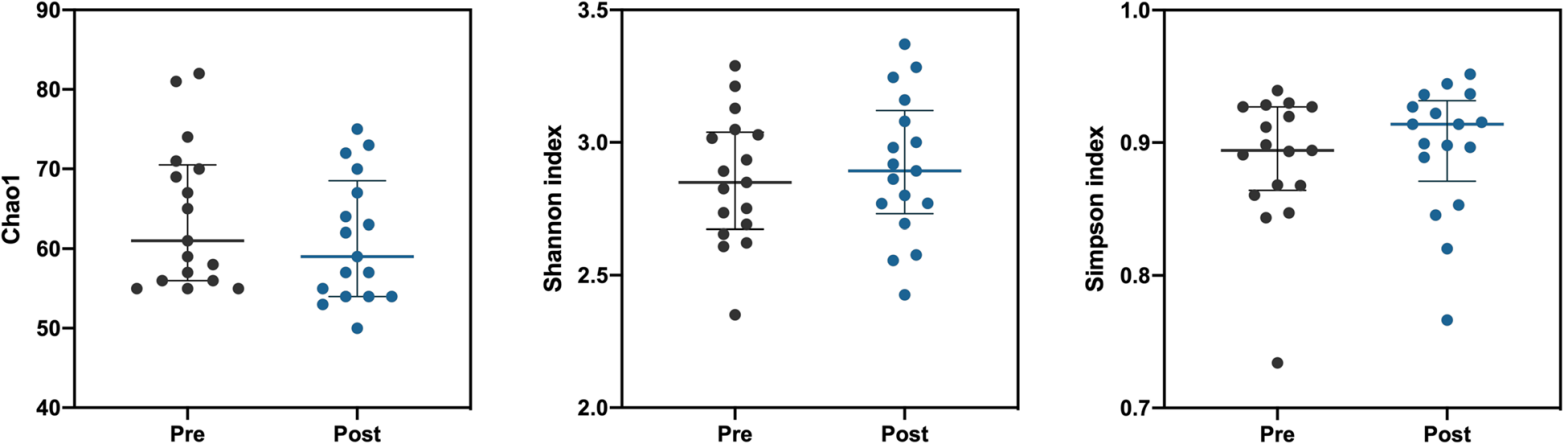
Comparisons of Chao1 index (left), Shannon index (middle) and Simpson index (right) of microbial communities in pre and post-training conditions

By sequencing the V3-V4 region of the 16S rRNA gene of *Archaea* and *Bacteria* from 34 faecal samples collected from 17 participants, we identified 11 phyla, 22 classes, 34 orders, 62 families and 123 genera. Physical exercise induced changes in microbiota composition, at all taxonomic levels analysed (PERMANOVA for paired data, Bray-Curtis dissimilarity index; phyla: F _(1, 32)_=3.97, *p*=0.029; classes: F _(1, 32)_=3.39, *p*=0.033, orders: F _(1, 32)_=3.17, *p*=0.044, families: F _(1, 32)_=1.54, *p*=0.037, genera: F _(1, 32)_=1.46, *p*=0.015, species: F _(1, 32)_=1.38, *p*=0.007). Conversely, no differences were found between pre and post-training conditions for microbial community richness (Chao1: Wilcoxon test, V=105, *p*=0.06) or diversity (Shannon index: V=62, *p*=0.52; Simpson index: V=59, *p*=0.43). Data are presented in Fig. 1.

#### Phylum level

**Fig. 2:**
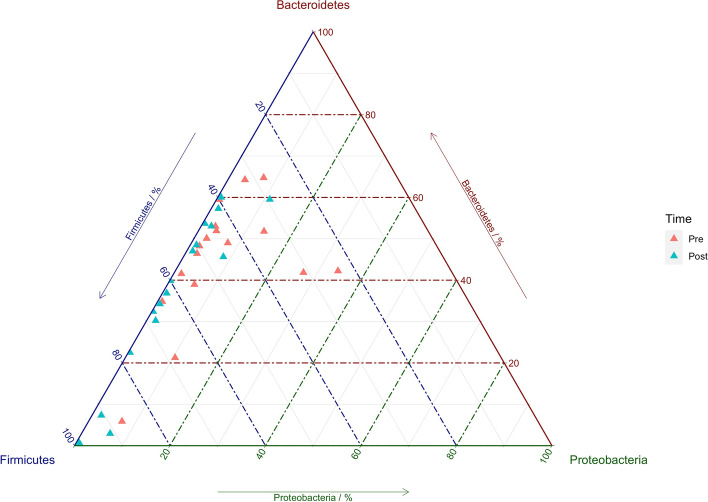
Ternary plot showing the distribution of samples in pre and post-training conditions, based on the relative abundance of three dominant phyla

The most abundant bacterial phyla identified were constituted by *Firmicutes*, *Bacteroidetes*, *Proteobacteria*: average relative abundances were 45.5% and 56.0% for *Firmicutes*, 43.5% and 35.3% for *Bacteroidetes*, and 7.6 and 2.2% for *Proteobacteria*, respectively in the pre and post-training conditions. The distribution of samples in the two conditions, based on the relative abundance of the three most dominant phyla, is represented in the ternary plot below (Fig. 2).

**Fig. 3:**
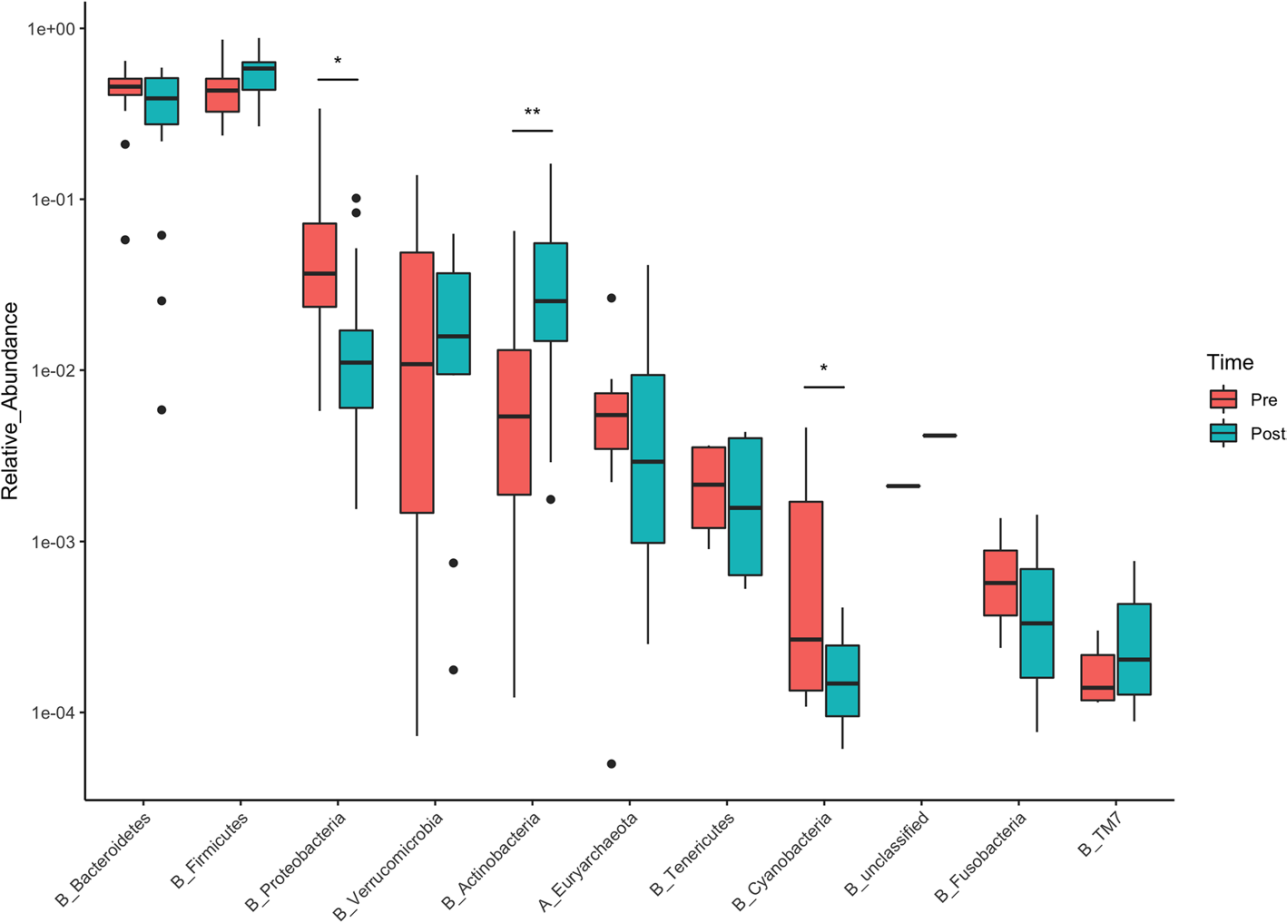
Box plots of relative abundances of phyla (expressed in logarithmic scale for visual clarity). Stars above the boxes represent statistical significance (*p<0.05, **p<0.01)

After checking for overall significance with the PERMANOVA test (see point 3.2), post-hoc univariate comparisons have been performed for all phyla with a Wilcoxon signed-rank test for paired data. Significant differences in relative abundances between post- and pre-training conditions were found for *Actinobacteria* (+3.38%; *p*=0.003, ES=0.72 (large effect)), *Cyanobacteria* (-0.05%; *p*=0.025, ES=0.54 (large effect)) and *Proteobacteria* (-5.45%; *p*=0.01, ES=0.614 (large effect)). Despite not reaching statistical significance, moderate effect sizes were found for *Bacteroidetes* (-8.28%; *p*=0.05; ES=0.47 (moderate effect)), and *Firmicutes* (+10.46%; *p*=0.08; ES=0.43 (moderate effect)). These results should be read keeping in mind the low sample size, which influences the significance of the tests. All the relative abundances of the phyla in pre and post-training conditions are reported in the box plots below (Fig. 3).

The *Firmicutes:Bacteroidetes* (F/B) ratio was calculated and compared in pre and post-training conditions. Median F/B ratio in pre-training was 0.89 (Q1: 0.67, Q3: 1.88), and in post-training was 1.50 (Q1: 0.85, Q3: 2.24) (Wilcoxon test, V=120, *p*=0.039, ES=0.50 (moderate effect)).

#### Genus level

As for phyla above, pre vs post-training comparisons were performed with a Wilcoxon test for paired data. A cut-off on the mean relative abundance of each genus was used, filtering data for an abundance equal or higher than 1%, in pre or post-training conditions. Twentynine genera were selected based on this criterion for further analyses. *Bifidobacterium*, *Parabacteroides*, *Prevotella*, *Dorea*, *Lachnospira*, *unclassified Ruminococcaceae*, *Phascolarctobacterium* and *Sutterella* were significantly different in post- vs pre-training. Descriptive (median (Q1 - Q3)) and test statistics for all the 29 genera are reported in Table 1. A list of all 123 genera comparisons is reported as an Additional file (Additional file 1).

**Table 1:** Changes in relative abundances of more represented genera (filtered for mean relative abundance >1% in pre or post-training condition). Differences between pre and post-training were tested using Wilcoxon paired tests. Effect sizes are also reported, with 95% confidence intervals

	Pre	Post	*p*	Effect Size (95% CI)
***Actinobacteria***				
*g_Bifidobacterium*	0.37 (0.16 - 1.27)	2.33 (1.41 - 4.91)	**0.004**	0.71 (0.37 - 0.88)
***Bacteroidetes***				
*g_Bacteroides*	32.3 (20.1 - 38.4)	21.1 (8.5 - 34.2)	0.163	0.34 (0.03 - 0.71)
*g_Parabacteroides*	2.43 (1.04 - 4.01)	1.39 (0.55 - 2.39)	**0.019**	0.57 (0.15 - 0.83)
*g_Prevotella*	0.42 (0.01 - 4.77)	0.03 (0.01 - 2.45)	**0.041**	0.48 (0.15 - 0.82)
*f_Rikenellaceae; g_*	2.86 (0.82 - 4.19)	3.18 (1.27 - 4.17)	0.523	0.15 (0.02 - 0.54)
***Firmicutes***				
*g_Lactobacillus*	0.00 (0.00 - 0.01)	0.00 (0.00 - 0.01)	0.866	0.05 (0.01 - 0.55)
*g_Streptococcus*	0.14 (0.07 - 0.48)	0.23 (0.14 - 1.01)	0.435	0.19 (0.01 - 0.67)
*g_Turicibacter*	0.18 (0.05 - 0.51)	0.19 (0.04 - 0.62)	0.586	0.13 (0.01 - 0.59)
*f_Clostridiaceae; g_*	0.38 (0.07 - 1.25)	0.27 (0.07 - 0.81)	0.723	0.09 (0.01 - 0.55)
*f_Lachnospiraceae; g_[Ruminococcus]*	0.88 (0.75 - 2.02)	1.07 (0.85 - 1.84)	0.381	0.21 (0.01 - 0.61)
*g_Blautia*	2.54 (1.78 - 3.14)	3.88 (1.83 - 9.50)	0.062	0.45 (0.07 - 0.77)
*g_Coprococcus*	2.15 (1.29 - 3.16)	2.73 (1.65 - 3.52)	0.113	0.38 (0.02 - 0.75)
*g_Dorea*	0.38 (0.16 - 0.62)	0.67 (0.37 - 1.82)	**0.019**	0.57 (0.06 - 0.88)
*g_Lachnospira*	0.84 (0.15 - 2.36)	0.41 (0.03 - 0.50)	**0.007**	0.65 (0.31 - 0.87)
*g_Roseburia*	2.70 (1.39 - 6.02)	1.81 (1.12 - 4.21)	0.227	0.29 (0.02 - 0.64)
*f_Lachnospiraceae; g_*	3.25 (1.09 - 4.17)	2.79 (2.28 - 4.64)	0.831	0.05 (0.00 - 0.51)
*f_Peptostreptococcaceae; g_*	0.97 (0.38 - 1.55)	1.08 (0.46 - 1.86)	0.593	0.17 (0.02 - 0.72)
*g_Faecalibacterium*	3.25 (2.54 - 4.21)	3.76 (2.99 - 6.41)	0.266	0.27 (0.02 - 0.65)
*g_Oscillospira*	1.73 (0.91 - 2.43)	2.08 (0.45 - 4.49)	0.246	0.28 (0.04 - 0.76)
*g_Ruminococcus*	0.91 (0.26 - 2.59)	1.73 (0.68 - 4.32)	0.055	0.46 (0.08 - 0.86)
*f_Ruminococcaceae; g_*	3.27 (1.98 - 6.06)	7.48 (4.73 - 8.36)	**0.003**	0.72 (0.36 - 0.88)
*o_Clostridiales; f_; g_*	0.83 (0.40 - 2.88)	0.60 (0.34 - 2.29)	0.493	0.17 (0.01 - 0.62)
*g_Dialister*	0.17 (0.01 - 3.17)	2.77 (0.00 - 3.46)	0.972	0.03 (0.00 - 0.51)
*g_Phascolarctobacterium*	0.37 (0.00 - 2.21)	0.03 (0.00 - 0.58)	**0.039**	0.55 (0.13 - 0.85)
*g_Veillonella*	0.03 (0.01 - 0.16)	0.00 (0.00 - 0.03)	0.084	0.39 (0.04 - 0.83)
*f_Erysipelotrichaceae; g_*	0.29 (0.12 - 0.77)	0.60 (0.23 - 1.15)	0.068	0.44 (0.05 - 0.78)
***Proteobacteria***				
*g_Escherichia*	0.46 (0.15 - 3.68)	0.13 (0.02 - 0.66)	0.062	0.45 (0.03 - 0.77)
*g_Sutterella*	0.71 (0.22 - 1.45)	0.15 (0.12 - 0.38)	**0.039**	0.50 (0.12 - 0.87)
***Verrucomicrobia***				
*g_Akkermansia*	0.00 (0.00 - 0.42)	0.07 (0.00 - 1.77)	0.767	0.08 (0.01 - 0.58)

**Fig. 4:**
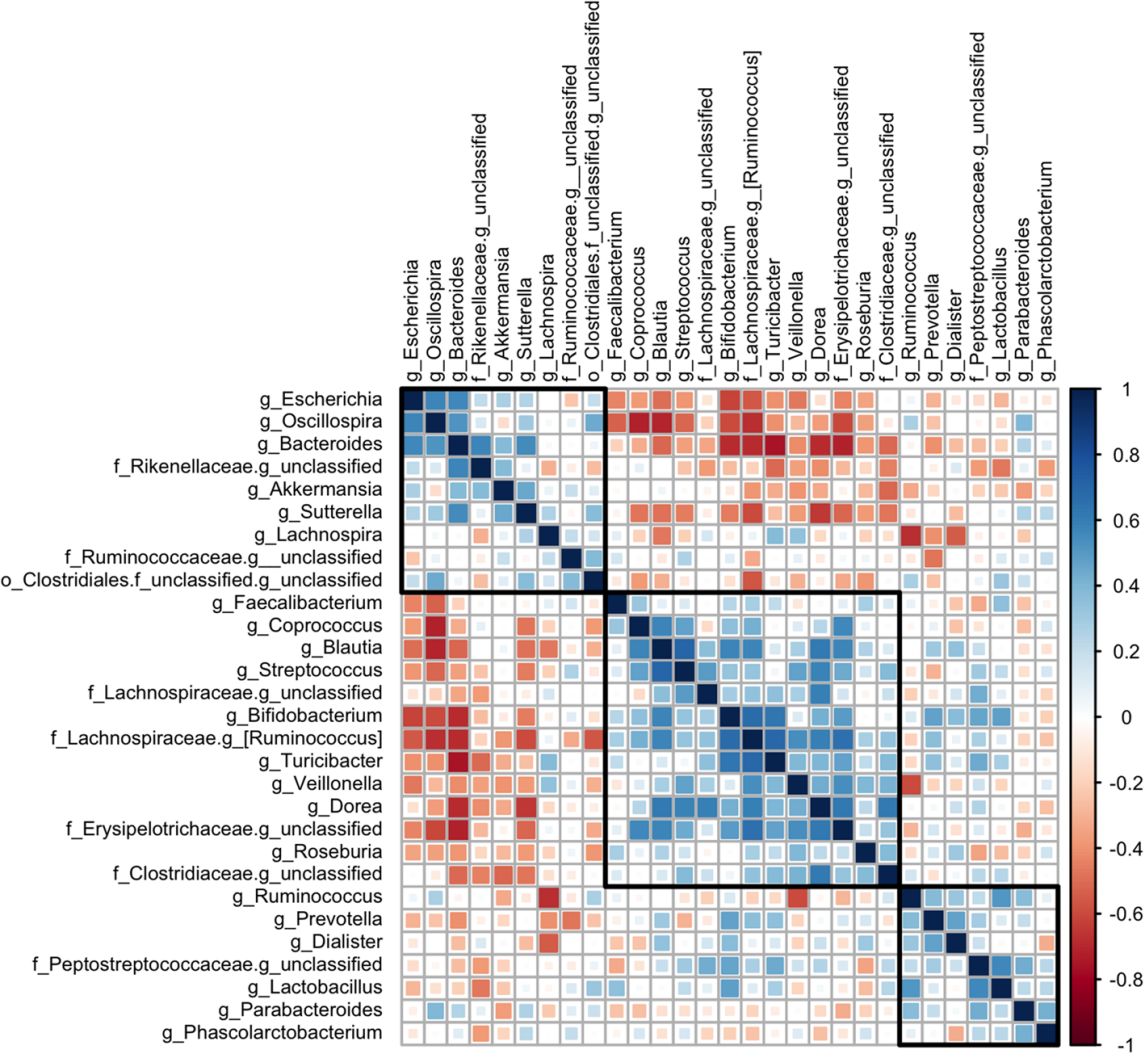
Correlation plots of delta (post-pre) at genus level: genera filtered for relative abundances over 1% in pre or post-training condition. Genera are ordered with hierarchical clustering (*hclust* method), and three subgroups of genera are highlighted

Non-parametric Spearman correlations of delta changes (post-pre) in the relative abundances of the 29 genera have been calculated to highlight subgroups of genera showing a similar change direction; correlations are presented in Fig. 4. Three clusters of genera can be identified: a first one (top left) is mainly composed of *Bacteroides*, *Lachnospira*, *Oscillospira*, *Sutterella*, *Escherichia* and *Akkermansia*; a second one (centre) in which the genera belonging to *Firmicutes* phylum (*Streptococcus*, *Turicibacter*, *Blautia*, *Coprococcus*, *Dorea*, *Roseburia*, *Faecalibacterium*, *Veillonella*) are prevalent, with the addition of *Bifidobacterium* (of the *Actinobacteria* phylum); finally, a third group (bottom right) formed by *Parabacteroides*, *Prevotella*, *Lactobacillus*, *Ruminococcus*, *Dialister* and *Phascolarctobacterium* can be highlighted. Interestingly, the genera of the first and second clusters are negatively correlated, while almost no correlation can be identified between the genera of the third cluster and the other two. A closer look at the clusters shows that most of the genera of the first cluster are reduced in abundance in post-training (except for *f_Ruminococcaceae.g_unclassified*), while the genera of cluster two show an opposite trend (except for *Roseburia* and *Veillonella*).

**Fig. 5:**
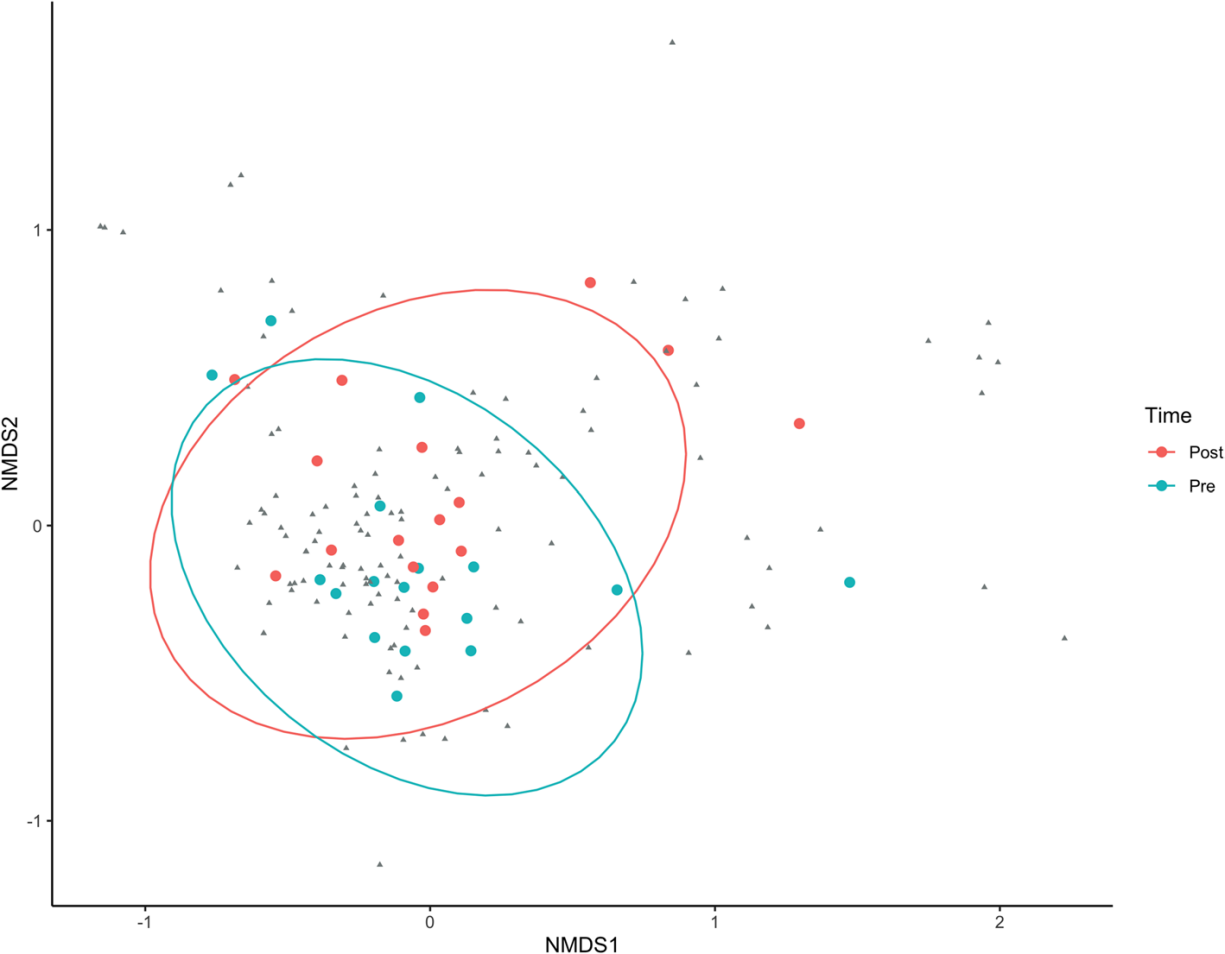
Non-metric dimensional scaling (PCoA) of Bray Curtis distance at the genus level, visualising the microbial composition in the pre- (light blue) and post-training (red) conditions

Non-metric multidimensional scaling (nMDS) is a method to explore and visualise dissimilarities of data matrix; it is a powerful tool to show specific patterns in multiple datasets. We used this approach to analyse the specific gut microbiota distribution patterns in pre-post training conditions. In Figure 5, a high dimensional space has been reduced into 2-dimensional space without too much loss of information (stress = 0.133, stress plot R^2^ = 0.98). Each bacterial genus was reported as small grey points, while subjects, in pre and post measurements, were reported in blue and red points in pre and post measurements, respectively. The ellipses referring to the pre-post conditions showed how some subjects had changed their position, while the majority fall into the common area of the two ellipses: From this, it can be inferred that, although PERMANOVA analysis showed a significant global variation (*p*<0.015), the training effect was noticeable only in a subgroup of subjects.

### Associations among changes in microbiota composition, dietary features and performance

The training period induced modifications in the subjects' physical composition, dietary habits, and performance indexes (Table 2). Even though assessing changes in these parameters was not among the aims of this study, these results must be described to allow a more straightforward understanding of the following associations among these and the changes in microbial composition. Nine weeks of high-intensity training induces a reduction of fat mass (-19.4%) and a gain of muscular mass (+3.7%), and an increase in relative peak power output (W_peak_/kg; +20.5%), and power at first (+45.2%) and second (+37.5%) lactate thresholds. In addition, as previously in a previous publication from our group (see Donati Zeppa et al. [14]), the training period promoted a spontaneous modulation of food choices. A significant increase in protein (+15.8%) and carbohydrate (+23.2%) intake has been observed during the training period. Energy and fats showed a similar trend, without a significant increase. A significant augment of fibres and vitamin C has been observed, suggesting higher consumption of fruit and vegetables during the training with respect to the pre-training.

**Table 2:** Environmental factors in pre and post-training. Mean ± SD, Δ% and t-value (Cohen's D Effect Size) are reported

Variable	Pre-training	Post-training	Δ%	t-value (ES)
**Physical features**				
Weight (kg)	68.1 ± 9.5	67.4 ± 7.5	- 0.6%	-1.182 (0.287)
Body Mass Index (kg/m^2^)	22.1 ± 2.6	22.1 ± 2.2	0.2%	-0.059 (0.014)
Fat Mass (%)	15.8 ± 5.4	12.5 ± 4.6	-19.4%	**-4.170 (1.011)**
Muscular Mass (kg)	42.1 ± 4.9	43.4 ± 3.8	3.7%	**2.708 (0.657)**
**Sport-related features**				
VO_2max_ (ml/min)	2965 ± 418	3133 ± 344	7.0%	1.935 (0.469)
VO_2max_ (ml/kg/min)	44.2 ± 8.0	46.5 ± 5.8	6.9%	1.723 (0.418)
W_peak_ (W/kg)	3.4 ± 0.4	4.1 ± 0.5	20.5%	**11.147 (2.703)**
LT_1_ (W/kg)	1.2 ± 0.3	1.7 ± 0.4	45.2%	**3.997 (0.969)**
LT_2_ (W/kg)	2.0 ± 0.5	2.7 ± 0.4	37.5%	**4.860 (1.179)**
**Dietary features**				
Energy (kcal)	2098 ± 481	2413 ± 790	16.2%	2.048 (0.497)
Protein (g)	84.4 ± 17.2	98.2 ± 30.0	15.8%	**2.642 (0.641)**
Carbohydrate (g)	266.9 ± 60.7	322.2 ± 100.9	23.2%	**2.548 (0.618)**
Starch (g)	129.7 ± 47.1	158.3 ± 61.9	40.5%	1.967 (0.477)
Fat (g)	78.9 ± 24.4	84.8 ± 36.3	9.4%	0.833 (0.202)
Saturated Fats (g)	23.0 ± 7.6	24.6 ± 10.2	10.1%	0.767 (0.186)
Monounsaturated Fats (g)	27.0 ± 9.3	28.5 ± 13.5	11.5%	0.517 (0.126)
Polyunsaturated Fats (g)	8.5 ± 2.9	10.0 ± 4.5	28.3%	1.152 (0.279)
Omega-3 (% kcal/kcal tot)	0.5 ± 0.2	0.4 ± 0.1	4.2%	-0.987 (0.239)
Omega-6 (% kcal/kcal tot)	3.1 ± 0.9	3.0 ± 0.5	5.8%	-0.201 (0.049)
Fibres (g)	8.4 ± 4.4	11.7 ± 8.4	48.4%	**2.203 (0.534)**
Iron (mg)	8.4 ± 2.6	10.0 ± 4.1	26.6%	1.982 (0.481)
Vitamin A (mcg)	772 ± 459	920 ± 540	44.1%	1.345 (0.326)
Vitamin C (mg)	48.7 ± 24.0	84.5 ± 51.7	117.4%	**3.150 (0.764)**
Vitamin D (mg)	1.7 ± 1.4	1.8 ± 0.7	54.9%	0.535 (0.130)
Vitamin E (mg)	7.0 ± 3.0	8.7 ± 4.9	29.7%	1.845 (0.448)
Aminoacids	12509 ± 4183	15121 ± 4916	28.6%	2.641 (0.641)

**Fig. 6:**
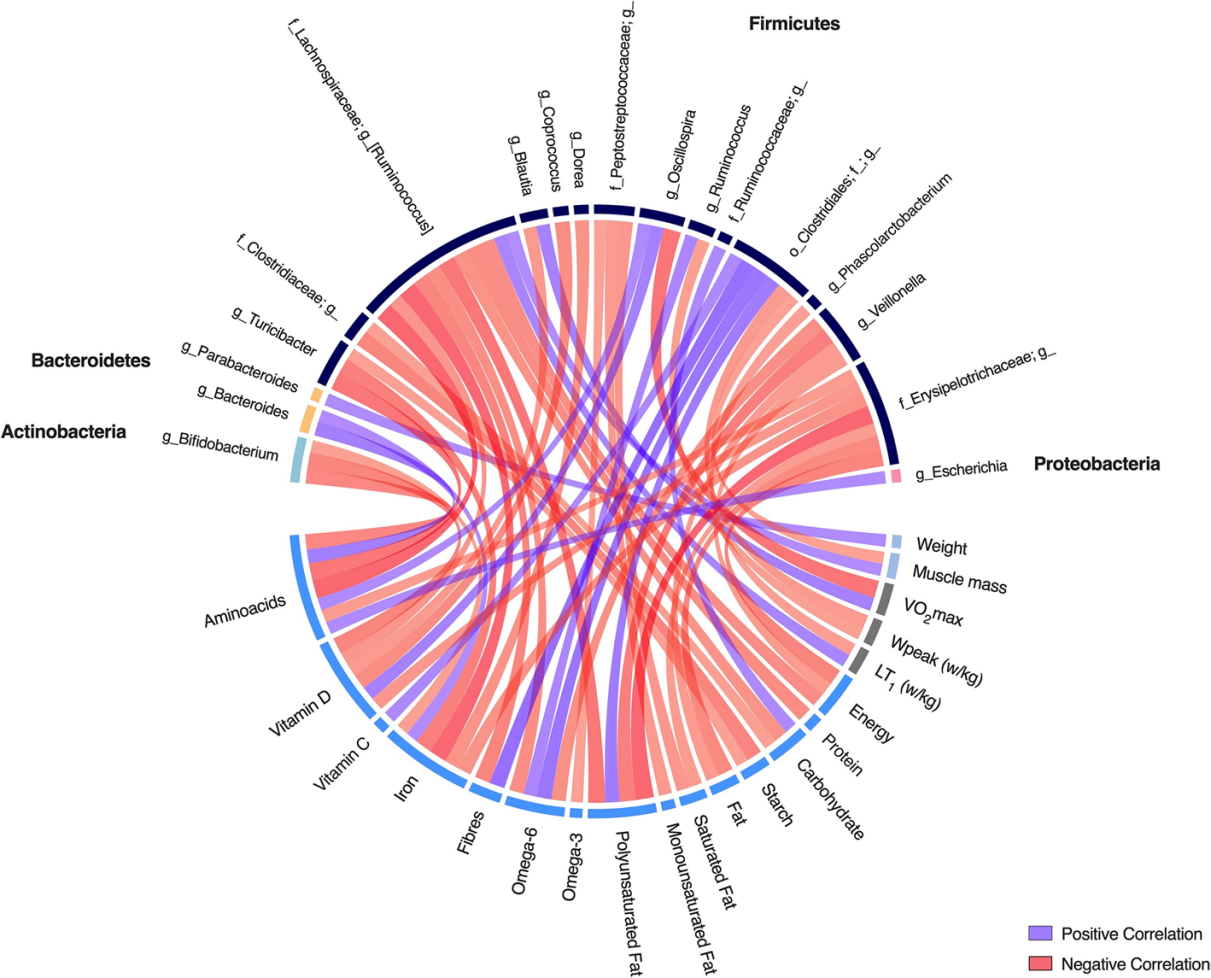
Spearman correlations between post-pre training delta of environmental factors and microbiota (genus level). Twenty-nine genera were selected for relative abundance >1%. 26 environmental factors were used for the correlation analysis, grouped in physical features [4], sport-related features [5], and dietary features [17]. Only significant correlations are reported in the figure. Sectors have different colours for groups of environmental factors and different phyla for simplicity. Purple links represent positive correlations, and red links represent negative correlations.

The correlations among the post-pre changes in the twenty-six factors presented in Table 2 and the bacterial taxonomic composition at the genus level (using the same cut-off of 1% in relative abundance as above) were investigated. Twenty factors and eighteen genera left after removing non-significant correlations (Spearman r ≥ 0.485, for n=17; p≤0.05, two-tailed), and the remaining are graphically presented as a circus plot in Fig. 6.

Among the physical features, a slight body mass reduction was accompanied by a decrease in abundance of *Parabacteroides* (r=0.50), while the muscle mass gain was associated with an increase of *[Ruminococcus]* (r=0.49) and a reduction of an unclassified genus of the *Clostridiales* order (r=-0.49). Among the indexes of performance, improvements in VO_2max_ and power at LT_1_ were positively associated with increased *Blautia* (r=0.55) and *[Ruminococcus]* (r=0.53), respectively. In contrast, VO_2max_ and LT_1_ were negatively correlated to *Oscillospira* (r=-0.69) and *Phascolarctobacterium* (r=-0.50), respectively. The increase in peak power output was negatively correlated with *Ruminococcus* (r=-0.50) and an unclassified genus of the *Clostridiales* order (r=-0.54). In addition, considering absolute values, without taking into consideration post-pre differences, *Bifidobacterium* was positively correlated with peak power output (r=0.34), while *Parabacteroides* was negatively correlated with peak power output (r=-0.38) and VO_2max_ (r=-0.37).

Among the dietary features, the increase in energy intake was negatively correlated with *[Ruminococcus]* (r=-0.59), *Veillonella* (r=-0.53) and an unclassified genus of the *Erysipelotrichaceae* family (r=-0.63). In addition, *unclassified Ruminococcus* showed negative correlations also with protein (r=-0.60), carbohydrate (r=-0.57), polyunsaturated fats (r=-0.69), omega-6 (r=-0.59), fibres (r=-0.63), iron (r=-0.72), vitamin D (r=-0.55), and amino acids (r=-0.74). Carbohydrate intake was also positively associated with an unclassified genus of *the Ruminococcaceae* family (r=0.51) and negatively correlated with an unclassified genus of the *Clostridiaceae* family (r=-0.50). All the correlations are reported as an Additional file (Additional file 2).

## Discussion

This study aimed to investigate changes in gut microbiota profiles, richness and diversity indices following nine weeks of training intervention and draw associations between microbial modifications, dietary habits, and sports performance changes. To our knowledge, this is the first training study that explored the effect of nine-week of high-intensity indoor cycling training on the microbiota composition in non-athlete healthy male college students, with a structured exercise period and daily monitoring of training and dietary intake. The results obtained suggest that this structured training induces partial changes to the relative abundance and community structure of gut microbiota, although no significant changes in *α*-diversity were found. The latter is in accordance with the results of Rettedal et al. [18], which to the authors' knowledge, is currently the only study in which the effects of a high-intensity training program on human gut microbiota were investigated. Indeed, the authors did not find any difference in *α*- or *β*-diversity after three weeks of HIIT in lean and overweight men. Other conflicting findings were reported by Yuan et al. [31] and Wang et al. [32], which found a lower and higher *α*-diversity (Simpson and Shannon indexes), respectively, after four and seven weeks of high-intensity exercise in mice. Greater microbiota *α*-diversity was reported in athletes [11], although no significant associations were reported between *α*-diversity and cardiorespiratory fitness levels [19].

### Microbial composition

Differences between pre and post-training conditions were detected at the phylum level, with an increase of *Actinobacteria* and a reduction of *Proteobacteria* and *Cyanobacteria*. No significant changes were found for *Firmicutes* and *Bacteroidetes*, although upward and downwards trends can be observed, respectively. Despite the absence of significance at the univariate level, a significant increase in *Firmicutes*/*Bacteroidetes* ratio (*F/B*) was identified after the training period. This result is in agreement with other cross-sectional studies, which reported higher levels of *Firmicutes* and lower levels of *Bacteroidetes* (thus an increased *F/B* ratio) in professional rugby players with respect to high-BMI controls [11] and adult’s elite respect to young elite and non-elite rowers [1]. *F/B* ratio was reported to be positively correlated with a higher VO_2max_ and with faecal total short-chain fatty acids [1]. An increase in the *F/B* ratio has also been observed by Huang et al. [33] in obese adolescents after six weeks of exercise intervention along with dietary restriction. *Firmicutes* are known to produce butyrate, considered a health-promoting molecule due to its role in regulating energy metabolism and increasing insulin sensitivity [34]. A decrease of *Proteobacteria* and *Cyanobacteria* and an increase of *Actinobacteria* have been observed after nine weeks of training in our study, suggesting the positive effect of exercise in promoting the host's health. Indeed, training promoted a decrease of the inflammatory process by reducing bacteria associated with pro-inflammation such as *Proteobacteria* and increasing bacteria involved in anti-inflammatory processes such as *Actinobacteria* [35]. In general, *Actinobacteria* - although they represent only a tiny percentage of the total microbial community - are key players in the maintenance of gut homeostasis, increasing tight junctions expression, regulating mucin biosynthesis and catabolism, providing energy for epithelial cells proliferation and stimulating the immune system [36]. Classes of this phylum, especially *Bifidobacteria*, are widely used as probiotics demonstrating beneficial effects in many pathological conditions, even if larger in vivo studies are needed to confirm such encouraging results. Moreover, *Bifidobacteria* show a non-negligible production of lactate, which can be metabolised by a group of bacteria defined as "lactate utiliser" to produce butyrate [36, 37].

At the genus level, to the authors' knowledge, to date, only one study investigated microbiota changes related to high-intensity interval training: indeed, Rettedal et al. [18] did not find any association between the top 50 most abundant genera and cardiorespiratory fitness markers, suggesting that a short-term (three weeks) high-intensity interval training do not impact the overall composition of the gut microbiome. In our study, a decrease in *Parabacteroides* has been correlated with weight reduction after the training period, a result that is in accordance with previous findings by Karvonen et al. [38]. Together with other markers, such as *Bacteroides*, *Blautia*, *Alistipes*, *Romboutsia* and *Roseburia*, *Parabacteroides* were reported to represent a common feature in obese patients with different metabolic disorders [39]. In contrast, it is worth mentioning that the training program in our study tended to increase *Blautia*, typically reduced in obese patients and related to a health microbiota profile. Notably, the increase of short-chain fatty acids producing bacteria, including *Blautia* and *Allobaculum*, has been reported to alleviate inflammation, insulin resistance, and obesity by reducing the intestinal endotoxins into the blood [17]. The training period also induced an increase in the abundance of *the Dorea* genus, which was previously reported to be negatively associated with insulin resistance [40]. Reduction in *Prevotella* found in our study is at odds with the results of Petersen et al. [41], who found that *Prevotella* abundance was positively correlated with the weekly training volume of competitive cyclists. However, it should be noted that in cyclists with a training volume less than ten h/week, *Prevotella* relative abundance was about 0.15%, a value comparable to the one we found in our sample. Furthermore, the *Sutterella* genus, previously identified as a common driver taxon in diabetes [42], showed a significant decrease after training. Lastly, the *Akkermansia* genus deserves mention, despite its slight increase after training did not result to be statistically significant. Indeed, *Akkermansia* was found to be one of the main exercise-responsive taxa in overweight women after six weeks of endurance training [16], and it has also been reported to be more abundant in male elite rugby players with respect to non-athletes [11].

### Nutrition

Diet is a major factor influencing the gut microbiome and should be considered a confounding factor when interpreting the results of an exercise intervention on microbiota composition. Some changes in the gut microbiota may be due to differences in dietary intake, in addition to the exercise itself [43]. Moreover, it has previously shown that exercise, particularly high-intensity exercise, might promote a spontaneous change of dietary choices toward a healthier direction [14]. So, to isolate and understand the direction of these relationships is not a foregone conclusion. Some differences or changes in the gut microbiota that seem to be associated with exercise might therefore be due to differences or changes in dietary intake, especially plants and carbohydrates, rather than the exercise itself. A significant increase in protein (+15.8%), carbohydrate (+23.2%) fibres, and vitamin C intake has been observed during the training period, suggesting higher consumption of fruit and vegetables during the training with respect to the pre-training. The mutual interactions between exercise, gut microbiota and diet, are the core in optimising performance and health. In particular, the increase of fibre intake leads to a higher production of short-chain fatty acids (SCFA), butyrate, propionate and acetate, which mediates the metabolic cross-talk between the gut microbiota and skeletal muscle [44]. SCFA affects muscle function, exercise capacity, glycogen accretion in skeletal muscle and performance [45, 46]. Furthermore, SCFA has a positive effect on muscle anabolism. In particular, butyrate and acetate stimulate glucose uptake, increase lipolysis and insulin sensitivity [47]. The effect of SCFA on skeletal muscles is mediated by activating muscular AMP kinase and the deposition of proteins in skeletal muscle tissue [48]. Furthermore, SCFA production is also associated with decreased inflammation and gastrointestinal disorders [49, 50].

In our study, several macro and micronutrient changes in post-pre training were associated with specific gut microbiota genera. Carbohydrate intake increases post-training and positively correlates with *Ruminococcaceae,* a commensal butyrate-producing bacteria belonging to the *Firmicutes* phylum, that can ferment indigestible carbohydrates and are crucial for maintaining health. *Bacteroides* affects amino acids’ availability and profile by participating in their digestion and absorption and are positively correlated with amino acids intake. SCFA can also be produced from branched-chain amino acids (valine, leucine, and isoleucine); threonine renders propionate and butyrate, whereas glutamate, histidine, lysine, arginine, and alanine give rise to acetate and butyrate formation. The metabolic versatility of *Bacteroides* and the modulation of its metabolism through the adequate balance of dietary proteins and carbohydrates could impact human health [51]. However, due to the major differences in population characteristics, dietary features, and data collection procedures, comparisons with other studies in the literature are complex and – in a certain sense – misleading.

### Performance indexes

The gut microbiome has been previously shown to be associated with fitness and performance measures, such as VO_2max_ and VO_2peak_. For example, either Allen et al. [13] and Estaki et al. [9] reported positive correlations among VO_2max_, VO_2peak_ and butyrate-producing bacteria. VO_2max_ has also been correlated with a higher *F/B* ratio [52, 53], although studies showed contrasting results [54]. Our study did not find any correlation between VO_2max_ and *F/B* ratio, but it should be considered that VO_2max_ values in our sample were quite homogeneous. Conversely, a negative correlation between VO_2max_ and *Parabacteroides* has been highlighted. Considering post-pre training changes, VO_2max_ showed a positive correlation with *Blautia* and a negative correlation with *Oscillospira*. A positive correlation between *Blautia* and VO_2peak_ was previously found by Yu et al. [54] in an elderly population. Relative power output at LT_1_ was instead correlated with *f_Lachnospiraceae [Ruminococcus]* and *Phascolarctobacterium*, positively and negatively, respectively. The former is a key butyrate-producing member of a family that has been shown to be more represented in aerobically fit individuals [9]. A study by Scheiman et al. reported a high abundance of *Veillonella atypica* in the post-exercise state in athletes after completing the Boston Marathon, suggesting a possible association with running performance [55]. In contrast, we did not find any change in *Veillonella* relative abundance after the training period. Although the mechanisms are still unknown, gut microbiota was suggested to have a bidirectional cross-talk with mitochondria during endurance exercise [45], and skeletal muscle bioenergetics might be modulated by exercise-induced alterations in microbiota composition [4, 56].

A limitation of this study is represented by the fact that it was not possible to deduce if microbial composition changes were related only to an exercise effect, to the changes in the dietary intake (possibly, exercise-driven) or a combination of both. A deeper understanding of this point should be of primary importance for future studies.

## Conclusions

Physical activity modifies the gut microbiota, exerting health benefits on the host; however, the specific bacteria associated with exercise are not yet known. This work showed that nine weeks of high-intensity indoor cycling training induced modifications in gut microbiota composition in non-athlete healthy male college students. Some relevant bacterial taxa were modified after the training period shifting the gut microbial population towards a healthier microbiome. Understanding the direction in which microbes play a key role in influencing athletic performance is of particular interest to athletes who aim to improve their results in competition. However, such knowledge could be of benefit to human health in general.

## Supplementary Information

**Additional file 1.**

**Additional file 2.**
